# Biliary atresia

**DOI:** 10.1186/1750-1172-1-28

**Published:** 2006-07-26

**Authors:** Christophe Chardot

**Affiliations:** 1 Service de chirurgie pédiatrique, Hôpital Cantonal Universitaire de Genève, Rue Willi Donzé 6, CH 1205 Geneve, Switzerland

## Abstract

Biliary atresia (BA) is a rare disease characterised by a biliary obstruction of unknown origin that presents in the neonatal period. It is the most frequent surgical cause of cholestatic jaundice in this age group. BA occurs in approximately 1/18,000 live births in Western Europe. In the world, the reported incidence varies from 5/100,000 to 32/100,000 live births, and is highest in Asia and the Pacific region. Females are affected slightly more often than males. The common histopathological picture is one of inflammatory damage to the intra- and extrahepatic bile ducts with sclerosis and narrowing or even obliteration of the biliary tree. Untreated, this condition leads to cirrhosis and death within the first years of life. BA is not known to be a hereditary condition. No primary medical treatment is relevant for the management of BA. Once BA suspected, surgical intervention (Kasai portoenterostomy) should be performed as soon as possible as operations performed early in life is more likely to be successful. Liver transplantation may be needed later if the Kasai operation fails to restore the biliary flow or if cirrhotic complications occur. At present, approximately 90% of BA patients survive and the majority have normal quality of life.

## Disease name

Biliary atresia

## Definition

Biliary atresia (BA) is a rare disease characterised by a biliary obstruction of unknown origin that presents in the neonatal period [1].

## Background

BA is the most frequent surgical cause of cholestatic jaundice in neonates. The common histopathological picture is one of inflammatory damage to the intra- and extrahepatic bile ducts with sclerosis and narrowing or even obliteration of the biliary tree [2]. Untreated, this condition leads to cirrhosis and death within the first years of life. Surgical treatment usually involves an initial attempt to restore bile flow: the Kasai portoenterostomy [3], which is performed as soon after diagnosis as possible. Later, liver transplantation may be needed if the Kasai operation fails to restore the biliary flow or if cirrhosis complications occur [4]. BA remains the most common indication for paediatric liver transplantation worldwide.

## Epidemiology

The reported incidence of BA varies from 5/100,000 live births in The Netherlands [5], 5.1/100,000 in France [6], 6/100,000 in the British Isles [7], 6.5/100,000 in Texas [8], 7/100,000 in Victoria Australia [9], 7.4/100,000 in Atlanta USA [10] and in Japan [11], 10.6/100,000 in Hawaii [12], to 32/100,000 in French Polynesia [13]. Incidence of BA is highest in Asia and the Pacific region. Females are affected slightly more often than males. Although some studies of time- and space-time distribution of BA cases have suggested seasonal variation and clustering of cases [8-10], these results have not been confirmed by larger studies [5,6], [14,15].

## Anatomical forms

Two different forms of BA have been identified [16]:

• Syndromic BA (~10%), associated with various congenital anomalies such as polysplenia, asplenia, cardiac or intra abdominal defects (situs inversus, pre-duodenal portal vein, absence of retro-hepatic inferior vena cava, intestinal malrotation).

• Non-syndromic BA (~90%), in which BA is an isolated anomaly.

Several surgical classifications of BA have been proposed. The French classification is based on the anatomical pattern of the extrahepatic biliary tract remnant (Table 1) [17,18].

**Table 1:** Anatomical types of biliary atresia (BA)

**French classification**	**Frequency**	**Description**	**Upper level of obstruction of the extrahepatic bile ducts**	**US/UK/Japanese classification**
Type 1	~3%	Atresia limited to the common bile duct	Common bile duct	Type 1
Type 2	~6%	Cyst in the liver hilum communicating with dystrophic intrahepatic bile ducts	Hepatic duct	Type 2
Type 3	19%	Gallbladder, cystic duct and common bile duct patent	Porta hepatis	Type 3
Type 4	72%	Complete extrahepatic BA	Porta hepatis	Type 3

## Aetiology

The aetiology of BA remains unknown. Some cases seem to be related to abnormal morphogenesis of bile ducts occurring early in gestation, while others appear to arise from later damage to normally developing bile ducts.

There are several strands of evidence to suggest that even in non-syndromic BA, the onset takes place early in gestation. Antenatal ultrasonography allows detection of those forms of BA that show cystic changes [19]. In a series of 10 infants detected antenatally, most were non-syndromic and the first abnormal scans were observed at about 20 weeks of gestation [20]. In one study on serial digestive enzyme sampling in amniotic fluid, gamma-glutamyl transpeptidase (gamma-GTs) levels were found significantly low as early as 18 weeks of gestation in infants born with non-syndromic BA, providing strong evidence of biliary obstruction at this term of gestation [21].

Human embryo studies have also revealed similarities between the appearance of developing bile ducts during the first trimester of pregnancy and the residual ductules seen at porta hepatis level in BA patients; thus, it was suggested that some cases of BA may result from alteration of the remodelling process of the bile ducts originating from the ductal plate membrane [22]. The persistence of primitive foetal-type bile ducts that leak bile into surrounding tissues and induce a secondary inflammatory reaction *in utero *has also been suggested. Recent studies have focused on normal and altered bile duct morphogenesis [23,24] and the initiation of hepatic fibrosis [25].

The role of viruses has been extensively studied. An association of BA with cytomegalovirus [26,27], respiratory syncitial virus [28], Epstein-Barr virus [29] and human papilloma virus [30] has been reported. In contrast, no association with hepatitis A, B and C viruses has been found [31,32]. Reovirus type 3 can cause cholangitis resembling BA in mice [33] and may be associated with spontaneous BA in the rhesus monkey [34]. In human neonates, the association of reovirus type 3 and BA has been suggested in several studies [35-38] but not supported in others [39-41]. Rotavirus type A can cause biliary obstruction in newborn mice, mimicking BA [42]; deleterious effects of rotavirus infection in mice can be prevented by interferon alpha [43]. In humans, the role of rotavirus type C in the aetiology of BA remains controversial [44,45].

Several observations suggest that a genetic component plays a role in the pathogenesis of BA, although this is probably only one of multiple factors. Familial cases of BA have been reported [46-50] although discordant sets of monozygotic twins have also been observed [51-53]. Variations in the incidence of BA among different races have been reported from Hawaii [12] and Atlanta, USA [10]. The incidence of HLA B12 and haplotypes A9-B5 and A28-B35 was found to be higher in infants with BA compared to a control group in one UK study [54].

## Clinical features

**Figure 1:**
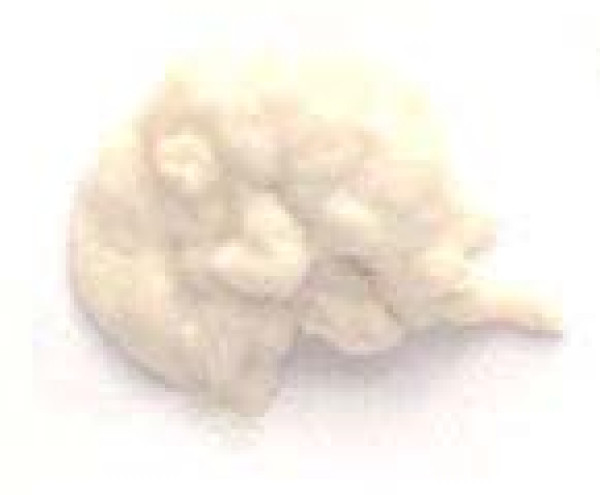
Acholic white stools.

After birth, the clinical triad of BA is:

• Jaundice (conjugated hyperbilirubinemia lasting beyond two weeks of life)

• Acholic (white) stools and dark urine (Figure 1)

• Hepatomegaly

The general condition of the child is usually good. There is no failure to thrive, at least in the first months. Thereafter, weight loss and irritability develop, accompanied by increasing levels of jaundice. Later signs include splenomegaly (suggesting portal hypertension), ascites and haemorrhage (which can be intracranial, gastrointestinal or from the umbilical stump) due to impaired absorption of vitamin K. Untreated, this condition leads to cirrhosis and death within the first years of life.

## Diagnosis and diagnostic methods

Since early diagnosis appears essential for effective surgical treatment [17,18], [55-57], every case of neonatal jaundice lasting more than two weeks should be investigated and biliary atresia actively excluded [58,59]. Diagnosis is made on the basis of the clinical manifestations and is supported by the following diagnostic methods:

### • Ultrasonography

Ultrasonography of the liver is performed after 12 hours of fasting (with an IV dextrose infusion). BA is suspected when the gallbladder is shrunk despite fasting, when the liver hilum appears hyperechogenic ("triangular cord sign") or when there is a cyst at the liver hilum. There should be no evidence of bile duct dilatation. Syndromic BA infants may show other features such as multiple spleens, preduodenal portal vein, absence of retrohepatic vena cava or abdominal situs inversus.

### • Cholangiography

When the gallbladder seems normal on ultrasonography scans, cholangiography is needed to assess the morphology and patency of the biliary tree. A cholangiogram can be obtained percutaneously (puncture of the gallbladder), endoscopically (ERCP) or at operation.

### • Liver biopsy

The main histological features suggestive of BA are bile plugs, ductular proliferation, portal oedema and/or fibrosis. As with any other cause of neonatal cholestasis, giant cell transformation may be observed.

### • Others

Biochemical liver function tests show cholestasis (with elevated cholesterol and gamma-GTs). Hepatobiliary scintigraphy (*e.g*. HIDA scans) demonstrates a failure of excretion of the radioisotope into the intestine, but this feature can also be observed in any severe neonatal cholestasis [60].

## Differential diagnosis

Medical causes of neonatal cholestasis must be excluded. The main differential diagnose are: Alagille syndrome, sclerosing cholangitis with neonatal onset, alpha-1-antitrypsin deficiency, cystic fibrosis, and more rarely progressive familial intrahepatic cholestasis (PFIC).

In most cases, diagnosis of BA can be strongly suspected after consideration of the clinical features, ultrasonography scans and exclusion of the main medical causes of neonatal cholestasis. Cholangiography and/or liver biopsy are indicated only in cases where the diagnosis remains uncertain, especially when the gallbladder seems normal on ultrasonography scans [61,62].

## Antenatal diagnosis

Antenatal diagnosis of BA remains exceptional. BA Types 1 and 2, which are rare, can be suspected on antenatal ultrasonography scans when a cystic structure is detected in the liver hilum [19,63]. Postnatal examination has to distinguish the cystic form of BA, which requires urgent surgery, from a choledocal cyst for which surgery may be delayed.

Non-visualization of the foetal gallbladder in early pregnancy (14–16 weeks gestation) may be associated with severe foetal anomalies, including polymalformation syndromes, chromosomal aberrations, cystic fibrosis [64]: amniocentesis is recommended for cystic fibrosis screening, hepatic enzymes tests and chromosomal analysis. Gallbladder may be visualised later in pregnancy, suggesting a delay in its recanalisation process. When the gallbladder remains undetectable after birth, the possibility that the patient has BA has to be carefully investigated. The incidence of agenesis of the gallbladder (without BA) is estimated at approximately 1/6000 pregnancies [64].

Features of polysplenia syndrome may be detected by antenatal ultrasonography. They may be part of a cardiosplenic syndrome whose prognosis depends mainly on the underlying cardiopathy [65,66]. Interrupted inferior vena cava may be isolated and benign [67]. However, neonates with features of the polysplenia syndrome should be carefully followed in order to rule out BA.

## Management

The current management of BA patients involves two steps:

• Kasai operation (in the neonatal period), which aims to restore bile flow.

• Liver transplantation in children for whom the Kasai operation has failed in its primary aim or for whom complications of biliary cirrhosis have supervened.

### • The Kasai operation: hepatoporto-enterostomy

**Figure 2:**
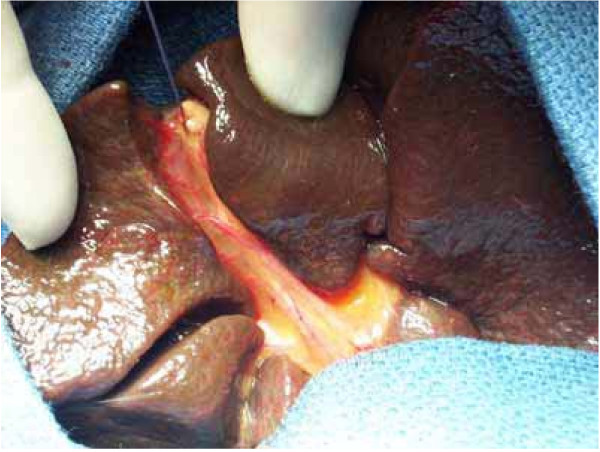
Operative view of complete extrahepatic biliary atresia.

**Figure 3:**
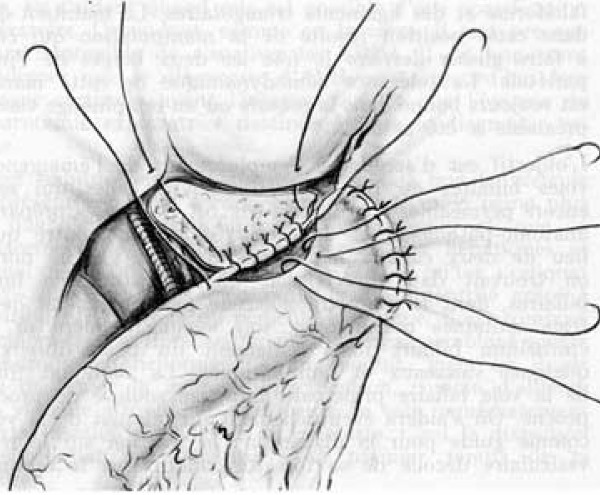
Hepatoporto-enterostomy (Kasai procedure) [68]*. * Valayer J, Chardot C: Atrésie des voies biliaires. Encycl Méd Chir (Elsevier SAS, Paris, All rights reserved), Techniques chirurgicales – appareil digestif (40–890); 2002: p. 12. Reproduction authorized by Elsevier Masson SAS.

After transverse supra-umbilical incision and laparotomy, diagnosis is confirmed by inspection of the liver and biliary tract. In most cases (type 4: complete extrahepatic BA), diagnosis is obvious with a cholestatic or fibrotic liver and a shrunken fibrotic gallbladder (Figure 2). If the gallbladder is still patent or if there is a cyst at the liver hilum, the colour of their content is noted and cholangiography is performed. Features of polysplenia syndrome, as well as any other intra-abdominal anomaly, are noted. The portal pressure can be measured through a small catheter introduced *via *the umbilical vein. After section of the falciform, left and right triangular ligaments, the liver is exteriorised out of the abdominal cavity. The entire extrahepatic biliary tree is excised together with the fibrous tissue occupying the space between the left and right branches of the portal vein at the level of the porta hepatis. A 45 cm Roux-en-Y loop is prepared and passed through the mesocolon to the liver hilum. An anastomosis is fashioned between the cut edge of the transsected tissue in the porta hepatis and the antimesenteric side of the Roux loop (Figure 3). A liver biopsy is performed.

**Figure 4:**
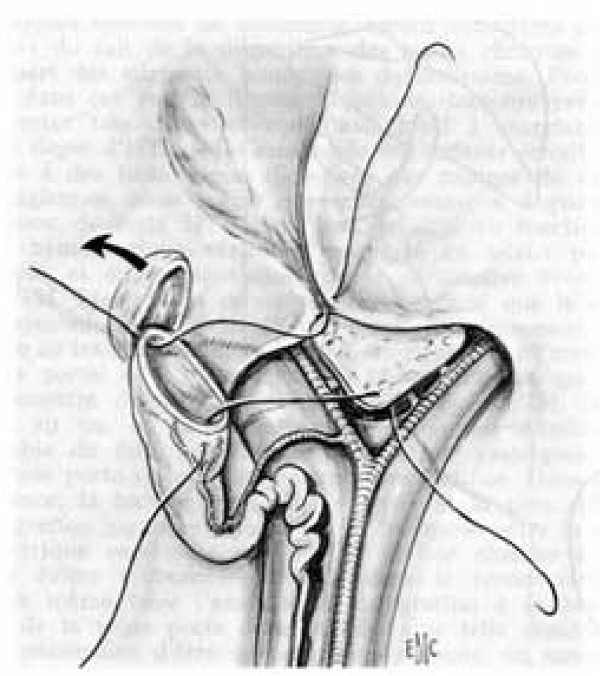
Hepatoporto-cholecystostomy [68]*. * Valayer J, Chardot C: Atrésie des voies biliaires. Encycl Méd Chir (Elsevier SAS, Paris, All rights reserved), Techniques chirurgicales – appareil digestif (40–890); 2002: p. 12. Reproduction authorized by Elsevier Masson SAS.

Many technical variants are possible, according to the anatomical pattern of the biliary remnant:

• Type 1 BA: cholecysto-enterostomy, or hepatico-enterostomy.

• Type 2 BA: cysto-enterostomy. This operation can be performed only if the hilar cyst communicates with the dystrophic intrahepatic bile ducts (as shown at cholangiography).

• Type 3 BA: hepatoporto-cholecystostomy. The patent galbladder, cystic duct and common bile duct are preserved. The gallbladder is mobilised with preservation of its pedicle. An anastomosis is performed between the gallbladder and the transsected tissue in the porta hepatis (Figure 4). Since there is no direct contact between the porta hepatis and the intestine, this operation reduces the risk of post-operative cholangitis [57]. Its specific complications, however, are bile leaks and post-operative biliary ascites caused by kinking and obstruction of the cystic duct and the common bile duct [69-71].

### • Post-operative course

Post-operatively, different drugs have been proposed either for reducing the inflammatory process at the liver hilum, which might lead to granulation and fibrous scar obstructing the biliary ductules, or for increasing the biliary flow. Although recommended by several surgeons [72-74], the use of corticosteroids remains controversial since their long-term benefit has not been proven; in addition there is a theoretical risk of exacerbating cholangitis. During the evaluation phase of biliary atresia, the infant's diet typically is not changed; post-operative breastfeeding is encouraged when possible, but an energetic supplementation may be required to obtain a 150 to 180 Kcal/kg/day intake. As long as cholestasis persists, supplementation in fat-soluble vitamins (ADEK) is needed.

## Outcome after successful Kasai operation

**Figure 5:**
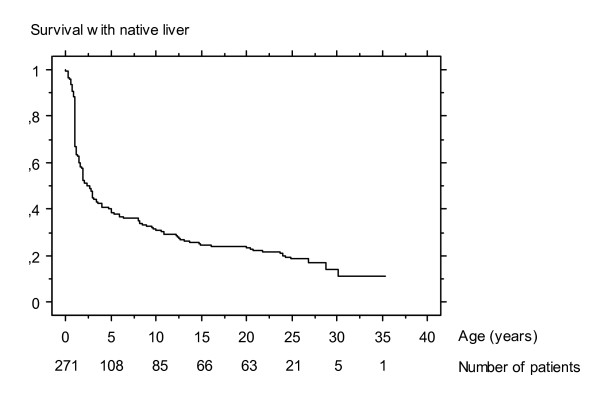
Survival with native liver of 271 infants who underwent Kasai operation for biliary atresia between 1968 and 1983 at Bicêtre hospital (Paris) [75]*. * Lykavieris P, Chardot C, Sokhn M, Gauthier F, Valayer J, Bernard O: Outcome in adulthood of biliary atresia: a study of 63 patients who survived for over 20 years with their native liver. Hepatology 2005, 41: p. 367. Reproduction authorized by John Wiley and Sons, Inc.

If the Kasai operation succeeds in restoring bile flow, the stools become coloured and jaundice fades. This process may last several weeks or months. The evolution of the biliary cirrhosis is prevented or at least delayed; survival with the native liver has been reported up to adulthood (Figure 5) [75,76].

The most common complications following the Kasai procedure include:

### • Cholangitis

Direct communication of the intestine with the dystrophic intrahepatic bile ducts, together with poor bile flow, can cause an ascending bacterial cholangitis. This occurs particularly in the first weeks or months after the Kasai procedure in 30%-60% of cases [77,78]. This infection may be severe and sometimes fulminant. There are signs of sepsis (fever or hypothermia, impaired haemodynamic status), recurrent jaundice, acholic stools and perhaps abdominal pain. Diagnosis can be confirmed by analysis of blood cultures and/or liver biopsies [78]. Treatment requires IV antibiotics and effective IV resuscitation. In the cases with recurrent and/or late cholangitis, obstruction of the Roux en Y loop or persisting colonisation of intrabiliary cyst should be considered. Recurrent cholangitis without a "surgical" cause may require continuous antibiotic prophylaxis.

### • Portal hypertension

Portal hypertension occurs in at least two-thirds of the children after porto-enterostomy [79,80], even in those with complete restoration of bile flow. The most common sites of varices include the oesophagus, stomach, Roux loop and anorectum. If the Kasai operation has clearly failed and the patient displays poor biochemical liver function and persisting jaundice, then liver transplantation is indicated. However, variceal sclerotherapy or band ligation before liver replacement may be necessary. In those cases with good liver function and absence of jaundice, endoscopic therapy may be the only treatment necessary [81,82]. Transjugular intrahepatic portosystemic shunts (TIPS) are rarely used for this indication due to the young age of the patients, the frequently observed hypoplasia of the portal vein and the possible development of intrahepatic biliary cavities [83]. Surgical portosystemic shunts are nowadays rarely indicated, especially when transplantation is available, but should be considered when there is a normal liver function, non-progressive liver disease and life-threatening varices [84]. Severe hypersplenism may exceptionally require splenic artery embolisation [85].

### • Hepatopulmonary syndrome and pulmonary hypertension

Similarly to patients with other causes of spontaneous (cirrhosis or prehepatic portal hypertension) or acquired (surgical) portosystemic shunts, pulmonary arteriovenous shunts may occur even after complete clearance of jaundice (hepatopulmonary syndrome). Gut-derived vasoactive substances that are not cleared by the liver (due to the portosystemic shunts) may be responsible for this complication. Typically, hepatopulmonary syndrome causes hypoxia, cyanosis, dyspnoea and digital clubbing. Diagnosis is confirmed by pulmonary scintigraphy. Pulmonary hypertension can occur in cirrhotic children and may provoke syncope or even sudden death. Diagnosis of pulmonary hypertension is suggested by echocardiography. Liver transplantation reverses pulmonary shunts [86] and can reverse pulmonary hypertension (especially when diagnosed at an early stage) [87].

### • Intrahepatic biliary cavities

Large intrahepatic biliary cysts may develop several months to years after the Kasai operation, even in patients with complete clearance of jaundice. These cavities may become infected and/or may compress the portal vein, requiring external drainage. Cystoenterostomy [88] or liver transplantation may eventually be required.

### • Malignancies

Hepatocarcinomas, hepatoblastomas [89] and cholangiocarcinomas [90] have been described in the cirrhotic livers of patients with BA, in childhood or adulthood. Screening for malignancy has to be performed regularly in the follow-up of patients who underwent a successful Kasai operation.

## Outcome after unsuccessful Kasai operation

If the Kasai operation fails to restore the bile flow, biliary cirrhosis progresses and necessitates liver transplantation. This is usually performed in the second year of life but may be necessary earlier (from 6 months of life) when there is a rapid aggravation of the liver disease. BA represents more than half of the indications for liver transplantation in childhood. Transplantation may also be required in those cases where recurrence of jaundice (secondary failure of the Kasai operation) or complications of cirrhosis (*e.g*. hepatopulmonary syndrome) occur despite an initially successful outcome after the Kasai operation.

There are two sources of liver grafts:

• Cadaveric donor: the graft is rarely a full size liver taken from a size-matched paediatric donor. More commonly, the graft consists of a left lobe (segments 2+3) or a left liver (2+3+4) obtained after reduction or splitting of an adult liver graft.

• Living-related donor: usually from one of the parents of the child.

Currently, patient survival at 5 and 10 years after liver transplantation is more than 80% [91-94]. In most cases, the quality of life of the transplanted patient is close to normal. Normal somatic growth pattern and physical, sexual and intellectual maturity are usually achieved [95-97].

## Overall outcome of BA patients

The overall prognosis of BA patients has improved since the early days of paediatric liver transplantation. Nowadays about 90% of BA patients may hope to survive (Table 2), with a normal quality of life for most of them.

**Table 2:** Current prognosis of BA in France and the United Kingdom.

	**France 1997–2002 (271 patients)**	**UK 1999–2002 (148 patients) [98]**
Overall 4-year patient survival	87%	89%
4-year survival with native liver after Kasai operation	43%	51%
4-year survival after liver transplantation	89%	90%

Several prognostic factors have been identified in BA patients. Some of them are related to characteristics of the disease (and cannot be altered): the prognosis of the Kasai operation is worse when BA is associated with a polysplenia syndrome [17,18,99]; when macroscopic obstructive lesions of extra-hepatic biliary remnant are diffuse (prognosis worsens from type 1 to type 4) [17,55-57]; when histological obliteration of the bile ducts (especially at porta hepatis) is more severe [100,101]; and when liver fibrosis is more extensive at time of the Kasai operation (102–107). Other prognostic factors are related to the management of BA patients and can be improved:

• the chances of success of the Kasai operation decrease when the age at Kasai operation increases [17,55,57,59]. It is, therefore, very important to diagnose BA early.

• accessibility to liver transplantation [108]: the mortality while waiting for a liver graft has recently decreased due to the development of surgical techniques that increase the availability of liver grafts: liver graft splitting [109], living related liver donation [110].

• experience of the centre managing BA patients [7,17,111]. This point led the British health authorities to centralise all BA patients from England and Wales in three paediatric liver units [98]. In France, a collaborative policy between centres was promoted and a national observatory of BA was created, in order to standardize therapeutic results nationwide and evaluate the results of this decentralized management.

## Conclusion

With the sequential treatment including the Kasai operation in the first weeks of life and, in case of failure of this procedure, secondary liver transplantation, 90% of patients with BA can nowadays survive, with a normal quality of life for most of them. Early diagnosis and treatment by an experienced team provides children with the best chance of survival.

## Links

French Observatory of Biliary Atresia: 

European Federation for Biliary Atresia Research: 

European Biliary Atresia Registry: 

Biliary Atresia Research Consortium: 
